# An MIP-Based PFAS Sensor Exploiting Nanolayers on Plastic Optical Fibers for Ultra-Wide and Ultra-Low Detection Ranges—A Case Study of PFAS Detection in River Water

**DOI:** 10.3390/nano14211764

**Published:** 2024-11-03

**Authors:** Rosalba Pitruzzella, Alessandro Chiodi, Riccardo Rovida, Francesco Arcadio, Giovanni Porto, Simone Moretti, Gianfranco Brambilla, Luigi Zeni, Nunzio Cennamo

**Affiliations:** 1 Department of Engineering, University of Campania Luigi Vanvitelli, Via Roma 29, 81031 Aversa, Italy; rosalba.pitruzzella@unicampania.it (R.P.); riccardo.rovida@unicampania.it (R.R.); francesco.arcadio@unicampania.it (F.A.); luigi.zeni@unicampania.it (L.Z.); 2 Moresense Srl, Filarete Foundation, Viale Ortles 22/4, 20139 Milan, Italy; a.chiodi@moresense.tech (A.C.); g.porto@moresense.tech (G.P.); 3 Department of Chemistry, Biology, and Biotechnology, University of Perugia, 06123 Perugia, Italy; simone.moretti@unipg.it; 4 Department of Food Safety, Nutrition and Veterinary Public Health, National Institute of Health, Viale Regina Elena, 299, 00161 Rome, Italy; gianfranco.brambilla@iss.it

**Keywords:** molecularly imprinted polymers (MIPs), surface plasmon resonance (SPR), plastic optical fibers (POFs), UV-curable optical adhesive, per- and polyfluorinated substances (PFASs), perfluorooctanoic acid (PFOA), persistent organic pollutants (POPs)

## Abstract

In this work, a novel optical–chemical sensor for the detection of per- and polyfluorinated substances (PFASs) in a real scenario is presented. The proposed sensing approach exploits the multimode characteristics of plastic optical fibers (POFs) to achieve unconventional sensors via surface plasmon resonance (SPR) phenomena. The sensor is realized by the coupling of an SPR-POF platform with a novel chemical chip based on different polymeric nanolayers over the core of a D-shaped POF, one made up of an optical adhesive and one of a molecularly imprinted polymer (MIP) for PFAS. The chemical chip is used to launch the light into the SPR D-shaped POF platform, so the interaction between the analyte and the MIP’s sites can be used to modulate the propagated light in the POFs and the SPR phenomena. Selectivity tests and dose–response curves by standard PFOA water solutions were carried out to characterize the detection range sensor response, obtaining a wide PFAS response range, from 1 ppt to 1000 ppt. Then, tests performed on river water samples collected from the Bormida river paved the way for the applicability of the proposed approach to a real scenario.

## 1. Introduction

In recent decades, the growing attention to environmental contaminants has underscored the need to develop increasingly advanced detection methodologies to ensure the safety of our ecosystems and human health. Among the concerning emerging compounds, per- and polyfluorinated substances (PFASs) represent a class of chemicals with significant environmental impact.

PFASs are a group of organic chemical compounds containing fully fluorinated aliphatic chains; that is, all the hydrogen atoms were replaced with fluorine in the aliphatic chain. Fluorination makes these molecules highly stable and resistant to chemical agents and environmental conditions [1].

Common examples of PFAS include perfluorooctanoic acid (PFOA) and perfluorooctanesulfonic acid (PFOS). Both are considered of growing concern due to their persistence in the environment and their potential impact on human health [2].

These substances have been used in various industrial and consumer applications, which has led to their widespread presence in different environments. Some of the common sources of PFAS include drinking water; non-stick coatings, such as those used in pans; stain- and water-resistant fabrics; firefighting foams; personal care products; and industrial waste, land, and water near places of the production or disposal of PFAS [3,4,5].

Their resistance to environmental degradation and their persistence in the environment can contribute to large-scale diffusion and severely impact human health.

Studies suggest that long-term exposure to PFAS can affect various systems in the human body, including the liver, immune system, endocrine system, and reproductive system [6]. In particular, exposure to PFAS has been linked to fertility problems and fetal growth alteration. Moreover, some studies have highlighted an increased risk of certain forms of cancer, particularly testicular and kidney cancer. However, PFASs’ impact on human health and well-being is still under investigation [7,8,9], as health and environmental authorities carefully monitor this situation and establish recommendations and exposure limits based on available scientific evidence. Nevertheless, concerns about environmental persistence and potential impact on human health have led to restrictions in their use and efforts to find safer alternatives [10].

In fact, since 2009, these substances have been listed in the Stockholm Convention, an international agreement, as persistent organic pollutants (POPs) [11]. To stop more exposures, all new uses and imports containing traces of PFAS are required to pass through preliminary testing by the Environmental Protection Agency (EPA) and specific restrictions are established to regulate these chemicals through detailed guidelines [12]. In 2020, for the purpose of reducing and eliminating their use, the European Commission imposed limits for use of PFOA, PFOS, and related substances (ECHA/NR/23/01), with a limit in the order of parts per trillion (ppt or ng/L) [13].

PFAS environmental monitoring involves a combination of measurements. Techniques used to detect the presence of these substances in water, soil, and air include sampling from contaminated environments such as rivers, lakes, aquifers, and soil, followed by a high-performance chromatographic and mass spectrometric analysis (HPLC-MS) [14,15] or gas chromatography (GC) [16]. Despite being currently the most widely used methods, these techniques have some drawbacks that need to be considered, such as the costs of instruments, operational complexity, and the need for highly specialized staff to run the analysis. All these factors often lead to increasing the overall cost of the analysis. Another issue to consider is the sample matrix: the material in which PFASs are present can influence the accuracy and precision of the analyses. Complex samples may require more intricate preparations or additional techniques to obtain accurate results, thus making the whole procedure more time-consuming.

Continual technological advancements aim to mitigate many of these challenges, improving ease of use, instrument robustness, and cost reduction. Based on these, some other studies proposed nano-sensors as an alternative to traditional analytical devices. Nano-sensors include three fundamental parts: a receptor that selectively binds to the analytes of interest, a transducer where the receptors are immobilized, and a measurement reader, which amplifies the signal and makes it readable and measurable data [17,18]. In this context, the scientific community has worked on developing new sensors for the detection and removal of PFAS, to guarantee maximum operational efficiency and compliance with legal limits. Some studies have proposed valid alternatives, e.g., electrochemical sensors exploiting specific chemical reactions with PFAS to generate measurable signals [19,20], impedance sensors [21], potentiometric detection with Ion-Selective Electrodes (ISEs) [22], and a photoelectrochemical sensor [23]. Faiz et al. [24] developed an optical sensor based on Fabry–Perot Interferometry (FPI) that exploits the variation in optical properties in the presence of PFAS. Other sensors proposed rely on fluorescence [25,26] and on a colorimetric analysis [27]. Cennamo et al. [28] developed a sensor that uses a plastic optical fiber (POF), which exploits the physical phenomenon of surface plasmon resonance (SPR). This optical technology is used with different receptors to detect perfluorinated chemicals at low concentrations. In particular, one type is a specific bioreceptor made up of a self-assembled monolayer of an antibody on the sensitive gold surface (limit of detection (LOD) equal to 240 ppt) [28]. Another type, to overcome the disadvantages of an antibody, is based on molecularly imprinted polymers (MIPs) [29,30].

In this direction, analytical detection is one of the most valid options to face the challenge of real-time environmental monitoring and among the molecular recognition techniques applicable to portable and nano-structured sensors, molecular imprinting is certainly the most advantageous.

MIP-based receptors present several advantages with respect to natural receptors, such as their selectivity, long-term stability, lower production costs, and greater reliability of large-scale replication [31]. To lower the LOD, a new sensor system was designed, one that exploits the multimodality of the POF. It is characterized by two chips, one that is microstructured and filled with specific MIP for PFOA and another one used to trigger the SPR phenomenon [32].

Despite the excellent results obtained with this new approach, great difficulty was found in terms of achieving large-scale reproducibility of the drilled platform. The present study aims to address this challenge through the implementation of an innovative sensitive D-shaped POF chip based on nanolayers. This research focuses on introducing and optimizing different nanolayers on the POF chip, to significantly improve the detection capability of PFAS in terms of wide-range and ultra-low-range concentrations via unconventional sensors via SPR methods. Polymer-based nanolayers represent an alternative that is more suitable for large-scale production and reproducibility, as they lack the disadvantages of the previous configurations, such as differences between microstructures [32]. Moreover, the instrumentation used to produce nanolayers is easier to operate, and more sensors can be manufactured simultaneously. Finally, MIP nanolayers have several advantages with respect to bulk MIPs, such as ease of template extraction and a more reproducible structure, as polymerization in the internal parts of the bulk polymer is a complex phenomenon and not so easy to reproduce in different batches.

In fact, the first POF (nanolayer-based chip) effective refractive index changes when the analyte–MIP binding occurs, thus altering the plasmonic resonance conditions that are triggered in the SPR–POF probe linked in series, as described in the first work in which this sensing strategy was used [33]. Through this approach, it was possible to combine both the chemistry of the materials, with the use of ultra-thin layers (nanolayers) of UV-curable optical adhesives and MIP on a planar D-shaped POF. In a first step, binding tests were performed in aqueous solutions to determine the chemical performance parameters of the proposed sensor system. Next, control tests were carried out in order to assess the selectivity. Finally, river water samples were tested to evaluate PFAS concentration in a real scenario, and the obtained results were then compared to those achieved by a gold-standard technique.

## 2. Materials and Methods

### 2.1. Chemical Reagents

Perfluorooctanoate ammonium salt (FPO-NH4), 2,2-azobisisobutyronitrile (AIBN), (Vinylbenzyl) trimethylammonium chloride (VBT), and 1H,1H,2H,2H-perfluorodecyl acrylate (PFDA) were purchased from Sigma-Aldrich (Saint Louis, MO, USA) and additional purification was not performed. To remove stabilizers, ethylene glycol dimethacrylate, purchased from Sigma-Aldrich, was vacuum-distilled before use. The solvent was deionized water. FPO-NH4 was weighed and dissolved in ultrapure water (Milli-Q^®^, Merck KGaA, Darmstadt, Germany) to prepare stock solutions.

### 2.2. MIP and NIP Preparation

Using a previously established procedure [29], FPO-NH4 was chosen as the template molecule, while VBT and PFDA were used as functional monomers. Ethylene glycol dimethacrylate (EGDMA) was utilized as the cross-linking agent. The reagents were mixed according to the following molar ratios: template/VBT/PFDA/EGDMA—1:4:5:50. The mixture was sonicated until a visually uniform milky solution was produced, then deionized water was added to dissolve the chemicals (volume ratio of H_2_O:EGDMA = 1:17.5). Previously weighed AIBN was added to the solution in a non-stoichiometric ratio. Polymerization was thermally started at 74 °C.

In a similar way, a not-imprinted polymer (NIP) was developed without adding the PFOA (template) in the prepolymeric mixture.

### 2.3. SPR–POF Probe

Platform preparation was extensively described in the literature [34]. To summarize, a 1 mm diameter POF, made up by a 10 µm fluorinated cladding and a 980 µm PMMA core, was placed in a resin block, fabricated by a commercial 3D printer (Photon Mono X UV Resin SLA 3D Printer, Anycubic^®^, Shenzhen, China). The POF was then polished using two different types of polishing papers (5 µm and 1 µm grits) to remove the cladding and part of the core, attaining a characteristic D-shaped structure. The roughness of the exposed core is a key aspect that triggers the plasmonic phenomena. In particular, the polishing papers at 1 μm and 5 μm are appropriate for this phenomenon, exploiting an “8-shaped” pattern, as reported in [34].

**Figure 1:**
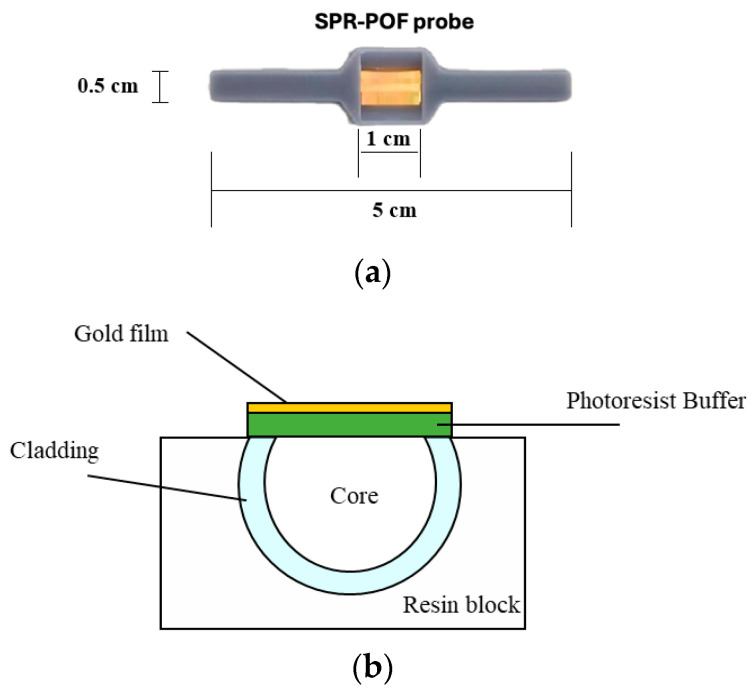
(**a**) A picture of the SPR–POF probe embedded in a 3D-printed holder. (**b**) A cross-section of the D-shaped POF plasmonic region.

Next, a layer of a photoresist buffer (thickness of about 1.5 µm) was deposited over the D-shaped fiber by spin-coating techniques. This buffer (Microposit S1813, produced by MicroChem Corp., Westborough, MA, USA) was characterized by a high refractive index (about 1.6 as shown in its datasheet) and it was used to enhance the plasmonic performance and to improve the gold adhesion [34]. Then, sputter-coating equipment (Safematic CCU-010, Zizers, Switzerland) was used to deposit a 60 nm gold nanofilm. Finally, the same resin used to fix the POF in the block was used to build a measuring cell. In this way, it was possible to use the cell to keep the liquid in contact with the SPR-POF chip for as long as necessary, thus satisfying the SPR condition. Figure 1a reports an actual image of the SPR-POF probe described above, whereas an outline of the cross-section plasmonic area is reported in Figure 1b.

### 2.4. Experimental Procedures for Liquid Chromatography–Mass Spectrometry (LC–MS/MS)

In total, 10 mL of the sample was spiked at 100 pg $g^{-1}$ with the twenty-one isotopically labeled internal standards (10 μL of a solution at 100 ng ${mL}^{-1}$) and then purified by means of weak anion exchange solid phase extraction (SPE). Phenomenex X-AW SPE cartridges (100 mg/3 mL, Phenomenex, Torrance, CA, USA) were conditioned with 3 mL of MeOH followed by 3 mL of water and rinsed three times with the eluting mixture (3 × 3 mL of 2% NH_4_OH in MeOH). The SPE cartridges were then re-conditioned again with 3 mL of MeOH and 3 mL of water. After sample loading, the cartridges were washed with 3 mL of water, 3 mL of MeOH 40%, and 6 mL (2 × 3 mL) of MeOH and dried under vacuum. Finally, the analytes were eluted with 3 mL of 2% NH_4_OH in MeOH into a 15 mL tube containing 80 mg of d-SPE Envicarb and 100 μL of acetic acid. After shaking and centrifugation (10 min at 12,000 rpm), the supernatant was transferred into a 15 mL tube with 50 μL of *n*-nonane. After evaporation near-dryness in a current of nitrogen (at 40 °C), 0.1 mL of 80:20 MeOH/ammonium acetate 4 mM was added. The solution was shaken manually and transferred into an autosampler vial. In particular, 40% methanol was used to remove the compounds retained by the SPE secondary interaction mechanism (reverse phase). Pure methanol was added to completely remove water from the column in order to reduce the evaporation time.

An instrumental analysis was performed following this as reported in Barola et al. [35].

## 3. POF Chemical Chips, Sensing Principle, and Experimental Setup

### 3.1. MIP-Based POF Chemical Chip

The POF chemical chip was prepared by fixing, into a 3D-printed holder, a 1 mm POF. The latter was then polished by two different lapping sheets with different grits, particularly a 5 µm grit one and then a 22 µm grit one (P800), hence obtaining the D-shaped POF area. The P800 polishing paper was used to improve the interaction of the light with the other polymer layers, similarly to [30].

Subsequently, a UV-curable optical adhesive (Norland Optical Adhesive 148, produced by Norland Products Incorporated, Jamesburg, NJ, USA), having a refractive index equal to 1.48, was deposited by a spin-coater on the D-shaped POF, in order to obtain a nanolayer as homogeneous as possible so as to anchor the MIP synthetic receptor. Over this nanolayer, the MIP prepolymeric mixture was deposited by spin-coating at a speed of 800 rpm, so as to produce an MIP nanolayer following the thermal polymerization step.

Then, the chemical chip was inserted into a specially designed 3D-printed cell to facilitate the incubation of standard solutions.

**Figure 2:**
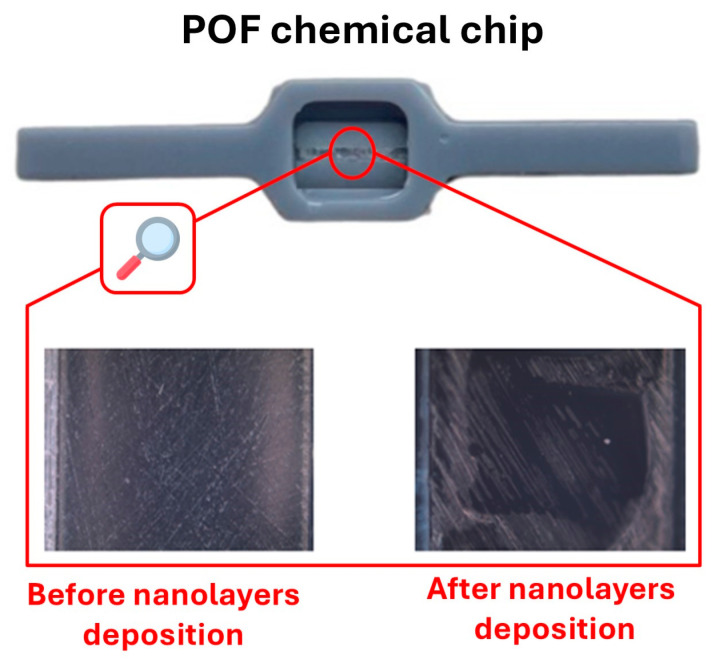
A picture of the MIP-based POF chemical chip embedded into a 3D-printed holder. Insets: The D-shaped POF surface before and after the nanolayer deposition.

A picture of the POF–chemical chip is shown in Figure 2. The insets in Figure 2 show two images of the modified POF acquired by an optical microscope (Axiotron microscope, Assy/Carl Zeiss, Oberkochen, Germany) before and after the nanolayer deposition steps. As it can be noticed, remarkable differences on the sensitive surface before and after the deposition of the nanolayers were achieved.

### 3.2. Sensor System and Sensing Principle

The sensor system is based on two different chips in series, i.e., the MIP-based POF chemical chip and the SPR-POF probe. A broad-spectrum white light source (model HL2000-LL, produced by Ocean Insight, Orlando, FL, USA) irradiated the MIP-based POF chemical chip, in which the analyte–receptor interaction takes place. The latter chip was used to launch the light into the following SPR-POF probe, on whose sensitive surface a fixed refractive index solution (n = 1.332) was deposited in order to trigger the SPR phenomenon. Finally, a spectrometer (model FLAME-S-VIS-NIR-ES, produced by Ocean Insight, Orlando, FL, USA) received the transmitted light from the SPR-POF probe. Then, the collected data were transmitted via a USB cable to a laptop for processing.

The sensing principle is the same as reported in [32,33] in order to improve the sensor sensitivity. More specifically, the binding between the analyte (PFAS) and the receptor (MIP) causes a variation in the effective refractive index of the optical waveguide (changes the refractive index of the MIP nanolayer) and a consequent variation in the mode profile of the light entering the SPR-POF probe. Therefore, the mode profile variation results in a shift in resonance wavelength that is not linked to changes in the refractive index of the medium in contact with the sensitive surface of the SPR-POF platform. In other words, the MIP–analyte interaction modulates the SPR conditions, thus producing a shift in the resonance wavelength [32,33].

**Figure 3:**
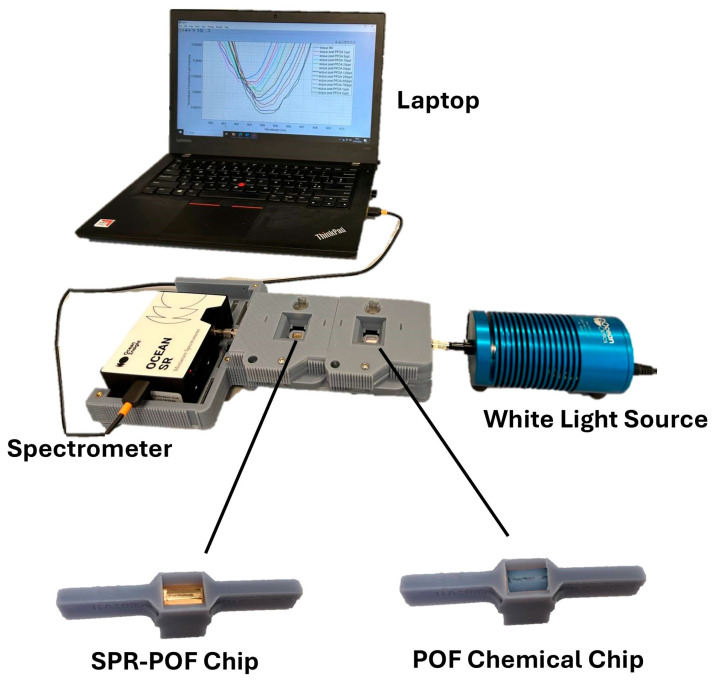
A picture of the experimental setup based on a custom 3D-printed holder, a white light source, two POF chips connected in series, and a spectrometer.

Figure 3 shows a picture of the experimental setup based on the equipment (white light source and spectrometer) and the custom holder manufactured using 3D printers. It was customized in such a way that the two sensors were connected via POFs, while the light source and the spectrometer were connected via subminiature assembly (SMA) connectors. The setup reported in Figure 3 is small-sized, portable, and low-cost, and can be connected to the Internet.

### 3.3. Measurement Protocol

The SPR spectra were obtained by normalizing the transmitted spectra on a reference spectrum. The latter was registered when air was considered as a surrounding medium on the SPR-POF probe (in this case, the SPR conditions were not fulfilled), while a blank solution (i.e., Milli-Q water) was present on the MIP-based POF chemical chip. Then, in order to trigger the SPR phenomenon on the plasmonic probe, 100 µL Milli-Q water was dropped onto the SPR-POF sensor (1.332), remaining fixed throughout the test. In particular, the 3D-printed holders for both the POF chemical chip and the SPR-POF chip had an integrated measuring cell (see Figure 3).

The binding tests were performed by dropping 100 μL of the solution under testing onto the MIP-based POF chemical chip-sensitive area. The SPR spectra were acquired after ten minutes of incubation with the standard solution and a washing step with Milli-Q water.

Thus, after the incubation and washing steps, the transmitted spectra were acquired with the fixed solution on the SPR-POF probe and the blank solution (i.e., water without analyte) present on the POF chemical chip [32].

### 3.4. Real-Sample Preparation

The real sample was collected from the Bormida river near the industrial plant located in Spinetta Marengo (Alessandria, Piedmont, Italy). The sample was diluted with MilliQ water, first at 1:1000 and then 1:500. Using the same protocol described in Section 3.3, the diluted samples were tested with the proposed sensor system.

## 4. Results

### 4.1. Dose–Response Curves in Milli-Q Water

Initially, the sensor system was tested for different concentrations of PFOA in MilliQ water. Solutions of PFOA were incubated (for 10 min) on the MIP-based POF chemical chip, and the refractive index (1.332) of the solution was fixed on the SPR POF probe [32,33]. The transmitted spectra were collected after the incubation and washing steps, with a blank solution over the POF chemical chip, as described in Section 3.3.

**Figure 4:**
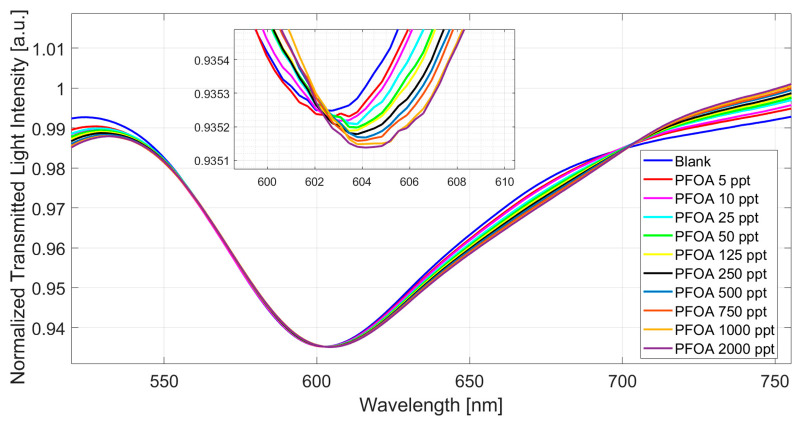
Normalized transmitted spectra (SPR spectra) achieved at different PFOA concentrations in MilliQ water ranging from 5 to 2000 ppt.

Figure 4 shows the SPR spectra obtained at increasing concentrations of PFOA in water in a concentration range from 5 ppt to 2000 ppt.

Figure 4 shows an increase (red shift) in the resonance wavelength value when the PFOA concentration increases. In particular, the MIP refractive index decreases when the PFOA concentration increases, and the propagated light mode profile changes the resonance conditions in the SPR-POF probe.

As shown in Figure 4, the sensor reaches saturation at about 1000 ppt. This response range is quite large, spanning three orders of magnitude. Such an aspect is quite important regarding possible applications, as many fields become available. From Figure 4 spectra, a data analysis was carried out using OriginPro software (version 9.2, Origin Lab. Corp., Northampton, MA, USA). The equation employed is the following:

(1) $$∆\lambda_{c}=\lambda_{c}-\lambda_{0}=∆\lambda_{max\;}\cdot \left(\frac{c^{n}}{K^{n}+c^{n}}\right)$$

where *c* is the PFOA concentration, $\lambda_{c}$ is the resonance wavelength at the concentration *c*, and $\lambda_{0}$ is the resonance wavelength of the blank, while ${\Delta \lambda }_{max}$ is the maximum value of ${\Delta \lambda }_{c}$, calculated with respect to the blank, attained at the saturation concentration. Δ*λ*_*max*_, *n*, and *K* are Hill constants.

**Figure 5:**
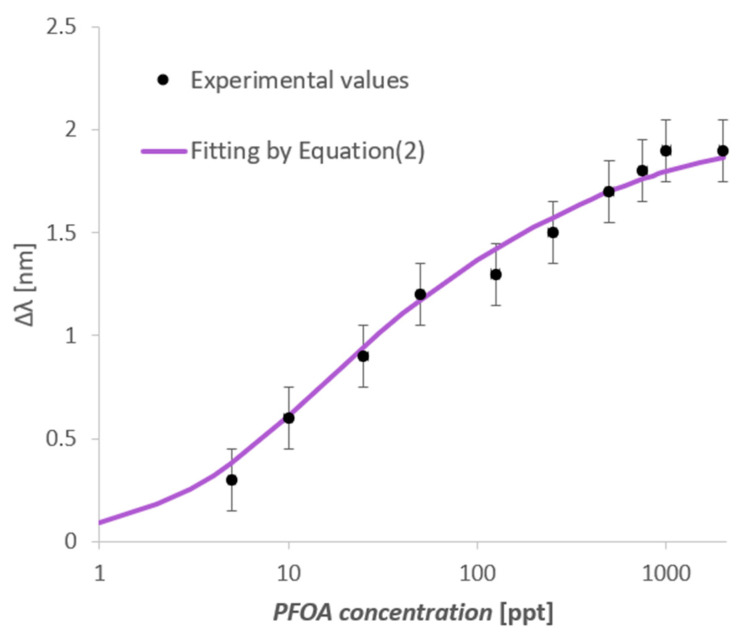
Resonance wavelength variations (calculated with respect to the blank) versus PFOA concentration in Milli-Q water, with the error bars and experimental fitting achieved by Equation (2), in a semi-log scale.

Figure S1 and Table S1 (Supplementary Materials) report the experimental values, relative to Figure 4, fitted by the Hill model and the corresponding fitting parameters, respectively. As shown in Table S1, considering the Hill model, the *n* parameter result is equal to 0.652, and thus significantly different from 1. In this case, the Langmuir model cannot be used because more specific sites with different affinity constants are present [36]. Thus, a second fitting model was used, in which two different kinds of sites are proposed with different affinities towards the analyte, one higher and one lower, as reported in Equation (2). The formation of sites with different affinities could be related to the deposition method employed in this case or the MIP shape. Consequently, the equation employed is the following:

(2) $$∆\lambda_{c}={∆\lambda }_{max,1}\cdot \;\frac{c}{K_{1}+c}+{\Delta \lambda }_{max,2}\;\cdot \;\frac{c}{K_{2}+c}\;$$

where ${\Delta \lambda }_{max,1}$ and $K_{1}$ are the parameters relative to the stronger-affinity sites (named as “sites1”), while ${\Delta \lambda }_{max,2}$ and $K_{2}$ are for the weaker-affinity sites (named as “sites2”). Figure 5 reports the dose–response curve achieved by Equation (2).

Considering that the sites work in different concentration ranges, the contribution of each kind of site was considered by setting n = 1 in the Hill model, which corresponds to the Langmuir equation for the adsorption isotherm of the template on the MIP for sites1 and sites2. In this case, the parameters *K*_1_ and *K*_2_ are the reciprocal of the affinity constants of the target molecule for the related imprinted site (strong or weak). For each type of site, when n = 1, at low concentrations, i.e., at *c*≪*K*, the related equation can be reduced to the linear relationship ${\Delta \lambda }_{c}={\Delta \lambda }_{max}\cdot c/K$, the slope of which is named as sensitivity at low concentrations; that is, $S_{low-conc}$=${\Delta \lambda }_{max}/K$.

In the Supplementary Materials, the Langmuir fitting for each kind of site is reported. In particular, Langmuir fitting for sites1 is shown in Figure S2, whereas Figure S3 is relative to sites2.

Table S2 in the Supplementary Materials lists the obtained parameters for both sites. The proposed multi-site approach properly fits the experimental data, thus denoting the presence of two sites with different affinity constants in the MIP receptor nanolayer.

For the stronger-affinity sites, sites1, the parameter *K* was 13.7 ppt, while for the weaker ones, sites2, it was 306.512 ppt. The affinity constant for both sites can be calculated as *K_aff_* = 1/*K*. At ultra-low concentrations, higher-affinity sites are filled first, while sites with lower affinity are occupied at higher concentrations.

Table 1 compares several affinity constants achieved via the same MIP receptor exploiting different sensor configurations. In particular, by comparing the affinities found with ones from previous work based on conventional SPR-POF-MIP sensors [29], it is possible to note that the affinity constant in [29]’s configuration is lower than sites1’s but similar to the one of sites2. The sensor configuration based on unconventional SPR reported in [32] presents an affinity constant similar to the one of sites1. In addition, compared with [32], the proposed novel sensor system via the second kind of affinity constant sites (sites2) can be used to achieve an ultra-wide detection range.

Table 1 reports, together with the affinity constants, other chemical parameters, such as the sensitivity at low concentrations and the detection limits.

For each kind of site, it is possible to calculate the LOD by the ratio between three times the standard deviation of the blank (for the proposed sensor reported in Table S2) and the *S_low−conc_*.

By comparing the LOD of the configuration of this study and the ones from previous studies, a few similarities can be highlighted. The stronger sites’ LOD and the one of the SPR-POF chip coupled with MIP-based chip configuration in [32] are similar, according to their affinity constants. However, the same can be said for the weaker sites’ LOD and the conventional SPR-POF-MIP configuration [29], as confirmed by the achieved affinity constants. The presence of two different types of sites is a strong advantage for this configuration, as one sensor can detect an ultra-wide detection range of analyte concentrations (from 5 to around 1000 ppts) while reaching saturation only at high concentration values. This aspect can be applied to a real-sample analysis and in situ analysis, both scenarios in which the analyte concentration is unknown and can significantly vary between sampling locations. For this purpose, a sensor that is able to respond in a wide concentration range is very useful.

### 4.2. Selectivity Tests

#### 4.2.1. Test on NIP-Based POF Chemical Chip

In order to test the selectivity of the proposed sensor system, POF chemical sensor chips with NIP nanolayers instead of MIP were developed and tested. These NIP-based POF chips were tested using the same protocol as the MIP-based POF chips described in Section 3.3.

**Figure 6:**
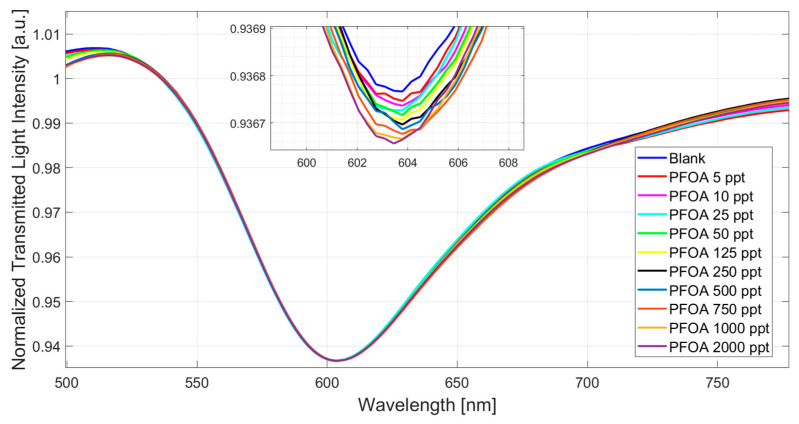
SPR spectra achieved at different PFOA concentrations in MilliQ water, ranging from 5 to 2000 ppt, via a NIP-based POF sensor configuration.

As Figure 6 shows, there is no significant shift in the resonance wavelength when the PFOA concentration is increased. This result shows that imprinting is primarily responsible for the specific interaction between the analyte and the different binding sites in the MIP.

#### 4.2.2. Selectivity Tests via MIP-Based POF Chemical Chip

**Figure 7:**
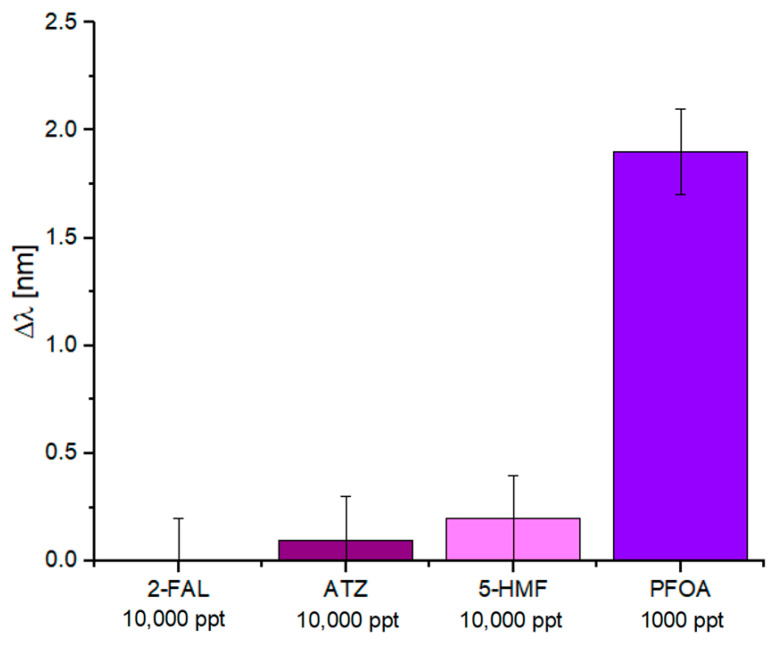
Selectivity tests: comparison between the resonance wavelength variations produced by 2-FAL, ATZ, and 5-HMF at 10,000 ppt and the analyte (PFOA) at 1000 ppt.

The selectivity of the MIP-based POF chemical sensor was tested with other substances in Milli-Q water. In particular, the substances chosen were 2-furancarboxaldehyde (2-FAL), Atrazine (ATZ), and 5-Hydroxymethylfurfural (5-HMF). Experimental results were carried out using the same measurement protocol described in Section 3.3. The concentrations of 2-FAL, ATZ, and 5-HMF (10,000 ppt) were an order of magnitude greater than the saturation value of the PFOA (1000 ppt). As shown in Figure 7, the other substances caused a slight shift even if they were 10 times more concentrated than the analyte solution. These variations could be ascribed to signal noise rather than a non-specific interaction produced by these chemicals. In other words, the other substances cause a slight shift, minor compared to 0.2 nm (the standard deviation value in the worst case), even if they are 10 times more concentrated than the analyte solution, and these variations can be considered in the error bar.

### 4.3. PFAS Detection in Real Samples

To investigate the capabilities of the developed PFAS sensor system, a real sample was analyzed using two approaches: exploiting the proposed POF sensor and the gold standard. More specifically, water from the Bormida river was collected near the industrial plant. The area around this industrial site is a well-known contaminated site by PFAS, as highlighted by recent studies performed by the Italian Agency for Environmental Protection (ARPA) [37]. Based on recent data, the regional government issued a new law establishing lower limits for PFAS detection [38]. In accordance with this new law, PFAS concentration must be in the ppt levels.

Blind measurements were performed, hence without any information about the expected concentration range.

Considering the ultra-low detection limit of the proposed sensor system, the sample was diluted several times, up to 1:1000, as described in Section 3.4, while the measurement procedure employed was the same as described in Section 3.3. The dilution steps can be used to achieve redundancy in estimating the concentration value and to reduce the non-specific interaction to prevent side effects on the sensor’s output related to the matrix complexity.

**Figure 8:**
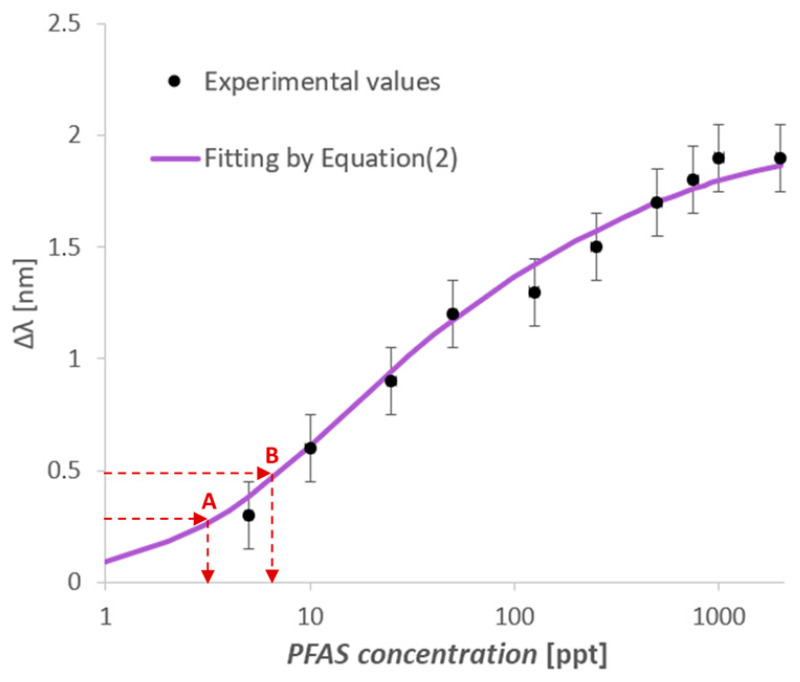
Estimated values of the PFAS concentration in the Bormida river via the sample at two dilution factors (1:1000, 1:500).

More specifically, the resonance shifts (Δ*λ*) relating to the different dilutions can be used on the dose–response curve to estimate the PFAS concentration in the tested real matrix samples, as shown in Figure 8. In fact, by considering the Δ*λ* values on the y-axis, achieved at different dilution factors (1:1000, 1:500), the corresponding concentration can be determined on the x-axis. Then, the PFAS concentration value is estimated by multiplying the concentration of the diluted sample by the dilution factor used, as summarized in Table 2 for points A and B.

The real sample from the Bormida river was tested using the LC-MS/MS technique as a gold standard. The procedure employed was described in Section 2.4, while the results are listed in Table 3.

The tested sample contains both legacy and emerging PFASs, as expected with a sample collected near a still-active industrial plant.

The total sum obtained from this analysis (gold standard) equals the one obtained from the proposed optical–chemical sensor system. It should be stressed that the MIP-based POF chemical chip exploits the same MIP synthesized in [29]. More specifically, the MIP is specific for PFAS detection in the range from C4 to C12, with the highest response from C8 PFAS [29]; thus, it is selective for all the PFASs present in the real sample, involving the fact that PFASs with different carbon chain lengths can interact with the sites in the polymer and bind themselves to those sites, generating a competing equilibrium between the template and similar substances.

Therefore, the MIP’s refractive index variation is due to the total sum of PFAS present in the sample. This result highlights that this approach is specific and selective towards PFAS, as the concentration value recorded was not linked only to PFOA but also to other similar substances present in the sample.

A direct speciation analysis of the kind of PFAS could be achieved via a different MIP receptor. Nevertheless, this aspect is not considered crucial for the sensor market, as the sensor can be employed for a wide-scope analysis on real scenarios outside simple laboratory use.

## 5. Discussion

The sensing technology used in this work is the result of the optimization of previously developed sensors [29,32]. In order to examine and evaluate the advantages and disadvantages of this sensing method, a comparative analysis was carried out between different methods for detecting PFAS already present in the literature. So, Table 4 shows the sensing principles of each kind of sensor, the target analyte, and the corresponding LODs.

Among the most studied and developed sensors, electrochemical ones, including voltammetric and potentiometric sensors, are the most common types used for PFAS detection [21,22,23]. Although electrochemical sensors are a promising option for PFAS detection, there are limits due to the materials used since they can be subject to deterioration over time due to phenomena such as the oxidation of the electrode material or contamination of the sensing surface. This could affect the long-term stability of the sensor and its reliability over time. Electrochemical sensors may also be susceptible to interference from other chemical species in the sample matrix. Consequently, sensor selectivity may be compromised in the presence of PFAS-like compounds. In fact, when testing these sensors [22] with contaminated waters from a New Jersey lake, it was observed that the system, capable of detecting low concentrations, could not discriminate between substances with similar structures that could interfere with the measurement. In this scenario, in addition to electrochemical sensors, fluorescent and optical sensors also play an important role. These sensors, based on fluorescence, offer an alternative or complement to electrochemical sensors, providing a different range of advantages and potential applications. Feng, H. et al. combine fluorescence with MIPs by imprinting them on the surface of SiO_2_ nanoparticles (NPs) [28]. This proposed method can selectively and sensitively detect up to 5570 ppt of PFOS in water. However, all fluorescent sensors are capable of detecting low concentrations of PFAS, but these types of sensors may be sensitive to environmental conditions such as temperature, pH, and solvent presence. Variations in measurement conditions can affect the stability of the fluorescence signal, compromising the reliability and reproducibility of measurements. Indeed, optical sensors offer the possibility to overcome the limitations of the mentioned technologies. This kind of probe, being inexpensive, flexible, durable, small-sized, and highly sensitive, allows for the design of portable sensors for accurate real-time analyses. Several studies have been conducted to date. For instance, one of the optical techniques utilized for developing PFAS sensors is based on FPI [24,39]. As an example, an FPI-based sensor reached an LOD of 400 ppt with regard to PFOA sensing in aqueous solutions. Despite offering considerable advantages, this advanced technique is sensitive to temperature and pressure, two parameters that can influence interferometric measurements.

Furthermore, it is expensive and requires specialized equipment, making the sensor unsuitable for an in situ analysis. Additionally, other types of optical sensors for PFAS detection have been developed so far. For the first time, Cennamo et al. [29] coupled an MIP with an SPR probe in POFs, already achieving better performance than antibodies. Given the reproducibility and robustness of the MIPs, a cascade configuration of two chips in series was subsequently investigated for the detection of PFOA concentrations in the ppt range [32]. However, the MIP-based POF chemical chip used in [32], based on three micro-holes achieving a D-shaped POF and filled by MIPs, denoted several limitations, mainly related to the reproducibility between different chips during the fabrication process. In this work, a different strategy was proposed to achieve the POF chemical chip to overcome these disadvantages. In fact, introducing and optimizing different nanolayers on D-shaped POF chips made it possible to improve the chemical chip’s reproducibility remarkably. This was possible since thin films could be produced on a large scale using controlled deposition techniques, allowing efficient and economical chip production. The employed deposition techniques are indeed easy to implement and lack the disadvantages of the previous configurations, such as differences between microstructures [32], making them more reproducible. Additionally, MIP nanolayers present a more reproducible structure with respect to bulk MIPs, other than an easier template extraction process.

**Figure 9:**
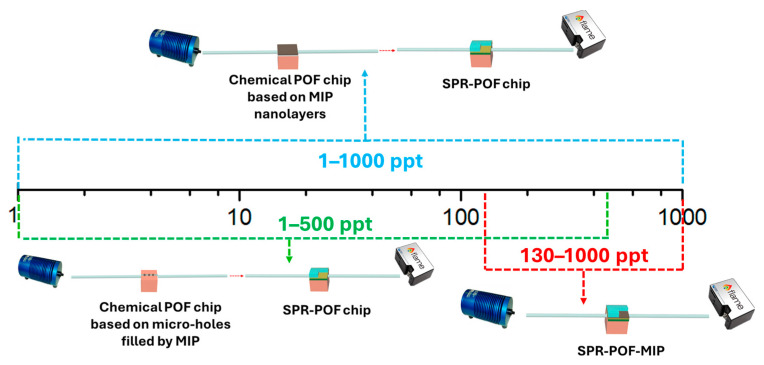
PFAS concentration ranges obtained by the same MIP receptor combined with different POF sensor configurations.

Finally, by exploiting two MIPs’ specific sites with different affinity constants present in the proposed sensor system, an ultra-wide detection concentration range can be obtained, covering the same concentration range achieved by the combination of [29,32] sensor configurations, as summarized in Figure 9.

The formation of ultra-thin films allowed greater control over the morphology of the material, which improved the selectivity of the sensor and reduced interference from other substances in the matrix [41].

## 6. Conclusions

A novel sensor tool for PFAS detection in a real scenario via a low-cost, portable, lightweight, and easy-to-handle device was developed and tested. The proposed sensor system presents an ultra-wide and ultra-low detection range from about 1 to 1000 ppt. The response range that is three orders of magnitude wide turns the proposed sensor into a useful tool, with possible applications to different in situ analysis scenarios, from environmental monitoring to known polluted site analyses. The sensor characterization was carried out using different experimental tests. In particular, PFAS detection was obtained via the proposed sensor system in a real sample from the Bormida river and cross-checked by an LC-MS/MS analysis from an external laboratory as a gold standard, confirming the POF tool’s capabilities for efficiently detecting PFAS compounds on site and in a few minutes, by a device connecting to the Internet via IoT approaches.

## Figures and Tables

**Table 1:** The comparison of the POF sensors’ parameters for PFOA detection exploiting sensor configurations based on the same MIP combined with different POF-based systems.

Sensor	*K_aff_* (*K_aff_* = 1/*K*) [ppt^−1^]	*S_low−conc_* [nm/ppt]	LOD [ppt]	Ref.
SPR-POF-MIP	0.005	0.022	130	[29]
SPR-POF chip + chemical POF chip based on micro-holes filled by MIP	0.036	0.063	0.81	[32]
SPR-POF chip + POF chemical chip based on MIP nanolayer sites1 (strong)	0.072	0.102	1.47	[present work]
SPR-POF chip + POF chemical chip based on MIP nanolayer sites2 (weak)	0.003	0.002	186.2	[present work]

**Table 2:** A summary of the estimated PFAS concentration in diluted samples from the Bormida River.

Label (Figure 8)	Dilution Factor	Δ*λ* [nm]	Estimated PFAS Concentration of Diluted Samples [ppt]	Estimated PFAS Concentration of Real Sample [ppt]
A	1:1000	0.3	4	4 × (1 × 10^3^) = 4 × 10^3^
B	1:500	0.5	8	8 × (5 × $\;10^{2})=4\;\times \;$ 10^3^

**Table 3:** Results of contaminated sample tested with LC-MS/MS technique, as average of three values.

Analyte	Concentration [ppt]—Linear	Concentration [ppt]—L + B
PFBA	460	466
PFPeA	423	646
PFHxA	134	151
PFHpA	149	164
PFOA	321	372
PFNA	137	150
PFDA	58	66
PFUdA	17	20
PFDoA	2	2
cC6O4	215	215
**Sum**	1916	2252
**Emerging PFASs**		
**Class**	**Compound**	**Concentration [ppt]**
ClPFPECA	ClPFPECA-0.1	581
	ClPFPECA-0.2	284
	ClPFPECA-0.3	132
	ClPFPECA-1.1	104
	ClPFPECA-1.2	11
	ClPFPECA-1.0 *	2
HPFPECA	HPFPECA-0.1 *	40
	HPFPECA-0.2 *	28
	HPFPECA-1.0 *	25
	HPFPECA-1.1 *	17
PFPEdCA	A2B2 *	140
	A2B3 *	61
	A3B *	77
	A3B2 *	73
	A4B *	40
	AB3 *	106
	B3 *	84
	B4 *	25
	**Sum**	1830
	**Total sum [ppt]**	4082

* Expressed as ClPFPECA-0.1.

**Table 4:** Comparative analysis in terms of LOD between different methods for PFAS detection.

Sensing Method	Target Analyte	LOD [ppt]	Ref.
Potentiometric detection using metal–organic framework and interdigitated electrodes	PFOS	0.5	[21]
Potentiometric detection using ISEs	PFO− a, PFOS	70–100	[22]
MIP-coated TiO_2_ nanotubes	PFOS	86	[23]
Ion-transfer stripping Voltammetry Fabry–Perot Interferometry (FPI)	PFCAs, PFSAs	25 × 10^3^	[39]
Graphene Oxide-Doped Alginate-Coated Optical Fiber Sensor (FPI)	PFOA	400	[24]
Aggregation-induced emission luminogens (AIEgen)	PFOA, PFOS, 6:2FTS	200	[25]
MIP fluorescence sensor	PFOS	5570	[28]
Self-assembled monolayer (SAM) on AuNP *	PFCs	25 × 10^4^	[40]
Electrochemical biosensor using an enzymatic biofuel cell (BFC)	PFOS	1.6	[20]
Antibody on SPR-POF	PFOA	240	[28]
SPR-POF-MIP	PFOA	130	[29]
Intensity-based POF-MIP	PFOA, PFOS	210	[30]
SPR-POF chip + chemical POF chip based on micro-holes filled by MIP	PFOA	0.81	[32]
SPR-POF chip + POF chemical chip based on MIP nanolayer sites1 (strong)	PFOA	1.47	This work
SPR-POF chip + POF chemical chip based on MIP nanolayer sites2 (weak)	PFOA	186.2	This work

* AuNP: Gold nanoparticle.

## Data Availability

The data presented in this study are available on reasonable request from the corresponding author.

## Supplementary Materials

The following supporting information can be downloaded at: https://www.mdpi.com/article/10.3390/nano14211764/s1, Figure S1: Resonance wavelength variations (calculated with respect to the blank) versus PFOA concentration in Milli-Q water with Hill fitting of the experimental values and error bars; Table S1: The Hill fitting parameters relative to PFOA detection; Figure S2: Resonance wavelength variations (calculated with respect to the blank) versus PFOA concentration in Milli-Q water with Langmuir fitting of the experimental values and error bars, relative to sites1 (strong); Figure S3: Resonance wavelength variations (calculated with respect to the blank) versus PFOA concentration in Milli-Q water with Langmuir fitting of the experimental values and error bars, relative to sites2 (weak); Table S2: The Langmuir fitting parameters relative to two different sites for PFOA detection.

## References

[1] Buck R.C., Franklin J., Berger U., Conder J.M., Cousins I.T., De Voogt P., Van Leeuwen S.P. (2011). Perfluoroalkyl and polyfluoroalkyl substances in the environment: Terminology, classification, and origins. Integr. Environ. Assess. Manag..

[2] Steenland K., Fletcher T., Savitz D.A. (2010). Epidemiologic evidence on the health effects of perfluorooctanoic acid (PFOA). Environ. Health Perspect..

[3] Stoiber T., Evans S., Temkin A.M., Andrews D.Q., Naidenko O.V. (2020). PFAS in drinking water: An emergent water quality threat. Water Solut..

[4] Brusseau M.L., Anderson R.H., Guo B. (2020). PFAS concentrations in soils: Background levels versus contaminated sites. Sci. Total Environ..

[5] Sima M.W., Jaffé P.R. (2021). A critical review of modeling Poly- and Perfluoroalkyl Substances (PFAS) in the soil-water environment. Sci. Total Environ..

[6] Pelch K.E., Reade A., Wolffe T.A., Kwiatkowski C.F. (2019). PFAS health effects database: Protocol for a systematic evidence map. Environ. Int..

[7] Bell E.M., De Guise S., McCutcheon J.R., Lei Y., Levin M., Li B., Ryu H. (2021). Exposure, health effects, sensing, and remediation of the emerging PFAS contaminants-Scientific challenges and potential research directions. Sci. Total Environ..

[8] Panieri E., Baralic K., Djukic-Cosic D., Buha Djordjevic A., Saso L. (2022). PFAS molecules: A major concern for human health and the environment. Toxics.

[9] Haervig K.K., Petersen K.U., Hougaard K.S., Lindh C., Ramlau-Hansen C.H., Toft G., Tøttenborg S.S. (2022). Maternal exposure to per- and polyfluoroalkyl substances (PFAS) and male reproductive function in young adulthood: Combined exposure to seven PFAS. Environ. Health Perspect..

[10] Bock A.R., Laird B.E. (2022). PFAS regulations: Past and present and their impact on fluoropolymers. Perfluoroalkyl Substances.

[11] Environmental Programme Stockholm Convention Ninth Meeting of the Conference of the Parties to the Stockholm Convention. Proceedings of the Ninth Meeting of the Conference of the Parties to the Stockholm Convention.

[12] EPA U.S. Drinking Water Health Advisories for PFOA and PFOS. https://www.epa.gov/ground-water-and-drinking-water/drinking-water-health-advisories-pfoa-and-pfos.

[13] PFASs Restriction Proposal—All News—ECHA—European Union. https://echa.europa.eu/-/echa-receives-PFASs-restriction-proposal-from-five-nationalauthorities.

[14] Gremmel C., Frömel T., Knepper T.P. (2017). HPLC-MS/MS methods for the determination of 52 perfluoroalkyl and polyfluoroalkyl substances in aqueous samples. Anal. Bioanal. Chem..

[15] Marra V., Abballe A., Dellatte E., Iacovella N., Ingelido A.M., De Felip E. (2020). A simple and rapid method for quantitative HPLC MS/MS determination of selected perfluorocarboxylic acids and perfluorosulfonates in human serum. Int. J. Anal. Chem..

[16] Casey J.S., Jackson S.R., Ryan J., Newton S.R. (2023). The use of gas chromatography-high resolution mass spectrometry for suspect screening and non-targeted analysis of per- and polyfluoroalkyl substances. J. Chromatogr. A.

[17] Nakamura H., Karube I. (2003). Current research activity in biosensors. Anal. Bioanal. Chem..

[18] Garg S., Kumar P., Greene G.W., Mishra V., Avisar D., Sharma R.S., Dumée L.F. (2022). Nano-enabled sensing of per-/poly-fluoroalkyl substances (PFAS) from aqueous systems-A review. J. Environ. Manag..

[19] Ranaweera R., Ghafari C., Luo L. (2019). Bubble-nucleation-based method for the selective and sensitive electrochemical detection of surfactants. Anal. Chem..

[20] Zhang T., Zhao H., Lei A., Quan X. (2014). Electrochemical biosensor for detection of perfluorooctane sulfonate based on inhibition biocatalysis of enzymatic fuel cell. Electrochemistry.

[21] Cheng Y.H., Barpaga D., Soltis J.A., Shutthanandan V., Kargupta R., Han K.S., Chatterjee S. (2020). Metal-organic framework-based microfluidic impedance sensor platform for ultrasensitive detection of perfluorooctanesulfonate. ACS Appl. Mater. Interfaces.

[22] Chen L.D., Lai C.-Z., Granda L.P., Fierke M.A., Mandal D., Stein A., Gladysz J.A., Bühlmann P. (2013). Fluorous membrane ion-selective electrodes for perfluorinated surfactants: Trace-level detection and in situ monitoring of adsorption. Anal. Chem..

[23] Li J., Feng H., Cai J., Yuan L., Wang N., Cai Q. (2014). Molecularly imprinted polymer modified TiO_2_ nanotube arrays for photoelectrochemical determination of perfluorooctane sulfonate (PFOS). Sens. Actuators B-Chem..

[24] Faiz F., Cran M.J., Zhang J., Muthukumaran S., Sidiroglou F. (2023). Graphene Oxide Doped Alginate Coated Optical Fiber Sensor for the Detection of Perfluorooctanoic Acid in Water. IEEE Sens. J..

[25] Fang C., Wu J., Sobhani Z., Al Amin M., Tang Y. (2019). Aggregated-fluorescent detection of PFAS with a simple chip. Anal. Methods.

[26] Feng H., Wang N., Yuan L., Li J., Cai Q. (2014). Surface molecular imprinting on dye-(NH2)-SiO_2_ NPs for specific recognition and direct fluorescent quantification of perfluorooctane sulfonate. Sens. Actuators B-Chem..

[27] Al Amin M., Sobhani Z., Liu Y., Dharmaraja R., Chadalavada S., Naidu R., Fang C. (2020). Recent advances in the analysis of per- and polyfluoroalkyl substances (PFAS)-A review. Environ. Technol. Innov..

[28] Cennamo N., Zeni L., Tortora P., Regonesi M.E., Giusti A., Staiano M., D’Auria S., Varriale A. (2018). A high sensitivity biosensor to detect the presence of perfluorinated compounds in environment. Talanta.

[29] Cennamo N., D’Agostino G., Porto G., Biasiolo A., Perri C., Arcadio F., Zeni L. (2018). A molecularly imprinted polymer on a plasmonic plastic optical fiber to detect perfluorinated compounds in water. Sensors.

[30] Cennamo N., D’Agostino G., Sequeira F., Mattiello F., Porto G., Biasiolo A., Zeni L. (2018). A simple and low-cost optical fiber intensity-based configuration for perfluorinated compounds in water solution. Sensors.

[31] Ahmad O.S., Bedwell T.S., Esen C., Garcia-Cruz A., Piletsky S.A. (2019). Molecularly imprinted polymers in electrochemical and optical sensors. Trends Biotechnol..

[32] Pitruzzella R., Arcadio F., Perri C., Del Prete D., Porto G., Zeni L., Cennamo N. (2023). Ultra-Low Detection of Perfluorooctanoic Acid Using a Novel Plasmonic Sensing Approach Combined with Molecularly Imprinted Polymers. Chemosensors.

[33] Cennamo N., Arcadio F., Zeni L., Alberti G., Pesavento M. (2022). Optical-Chemical Sensors Based on Plasmonic Phenomena Modulated via Micro-Holes in Plastic Optical Fibers Filled by Molecularly Imprinted Polymers. Sens. Actuators B-Chem..

[34] Cennamo N., Massarotti D., Conte L., Zeni L. (2011). Low cost sensors based on SPR in a plastic optical fiber for biosensor implementation. Sensors.

[35] Barola C., Moretti S., Giusepponi D., Paoletti F., Saluti G., Cruciani G., Brambilla G., Galarini R. (2020). A liquid chromatography-high resolution mass spectrometry method for the determination of thirty-three per- and polyfluoroalkyl substances in animal liver. J. Chromatogr. A.

[36] Pesavento M., Marchetti S., De Maria L., Zeni L., Cennamo N. (2019). Sensing by molecularly imprinted polymer: Evaluation of the binding properties with different techniques. Sensors.

[37] Polo Chimico di Spinetta Marengo: I Risultati Analitici in Seguito Agli Ultimi Eventi Accidentali. https://www.arpa.piemonte.it/news/polo-chimico-di-spinetta-marengo-i-risultati-analitici-in-seguito-agli-ultimi-eventi-accidentali.

[38] DECRETO LEGISLATIVO. 23 Febbraio 2023, n. 18. https://www.gazzettaufficiale.it/eli/id/2023/03/06/23G00025/sg.

[39] Garada M.B., Kabagambe B., Kim Y., Amemiya S. (2014). Ion-transfer voltammetry of perfluoroalkanesulfonates and perfluoroalkanecarboxylates: Picomolar detection limit and high lipophilicity. Anal. Chem..

[40] Niu H., Wang S., Zhou Z., Ma Y., Ma X., Cai Y.J. (2014). A Sensitive colorimetric visualization of perfluorinated compounds using poly (ethylene glycol) and perfluorinated thiols modified gold nanoparticles. Anal. Chem..

[41] Li Z., Lin Z. (2021). Two-dimensional polymers: Synthesis and applications. ACS Appl. Mater. Interfaces.

