# Supplementary material for: An MIP-Based PFAS Sensor Exploiting Nanolayers on Plastic Optical Fibers for Ultra-Wide and Ultra-Low Detection Ranges—A Case Study of PFAS Detection in River Water
Source: Nanomaterials (Basel). 2024 Nov 3;14(21):1764. doi: 10.3390/nano14211764 (PMC11547922; doi:10.3390/nano14211764)
Supplement: Supplementary file 1 [file nanomaterials-14-01764-s001.zip › nanomaterials-3267545-supplementary.pdf]

nanomaterials

MDPI

Supplementary Materials

An MIP-Based PFAS Sensor Exploiting Nanolayers on Plastic Optical Fibers for Ultra-Wide and Ultra-Low Detection Ranges—A Case Study of PFAS Detection in River Water

Rosalba Pitruzzella [1], Alessandro Chiodi [2], Riccardo Rovida [1], Francesco Arcadio [1], Giovanni Porto [2], Simone Moretti [3], Gianfranco Brambilla [4], Luigi Zeni [1] and Nunzio Cennamo [1,*]

[1] Department of Engineering, University of Campania Luigi Vanvitelli, Via Roma 29, 81031 Aversa, Italy; rosalba.pitruzzella@unicampania.it (R.P.); riccardo.rovida@unicampania.it (R.R.); francesco.arcadio@unicampania.it (F.A.); luigi.zeni@unicampania.it (L.Z.)
[2] Moresense Srl, Filarete Foundation, Viale Ortles 22/4, 20139 Milan, Italy; a.chiodi@moresense.tech (A.C.); g.porto@moresense.tech (G.P.)
[3] Department of Chemistry, Biology, and Biotechnology, University of Perugia, 06123 Perugia, Italy; simone.moretti@unipg.it
[4] Department of Food Safety, Nutrition and Veterinary Public Health, National Institute of Health, Viale Regina Elena, 299, I-00161 Rome, Italy; gianfranco.brambilla@iss.it
* Correspondence: nunzio.cennamo@unicampania.it; Tel.: +39-081-5010-379

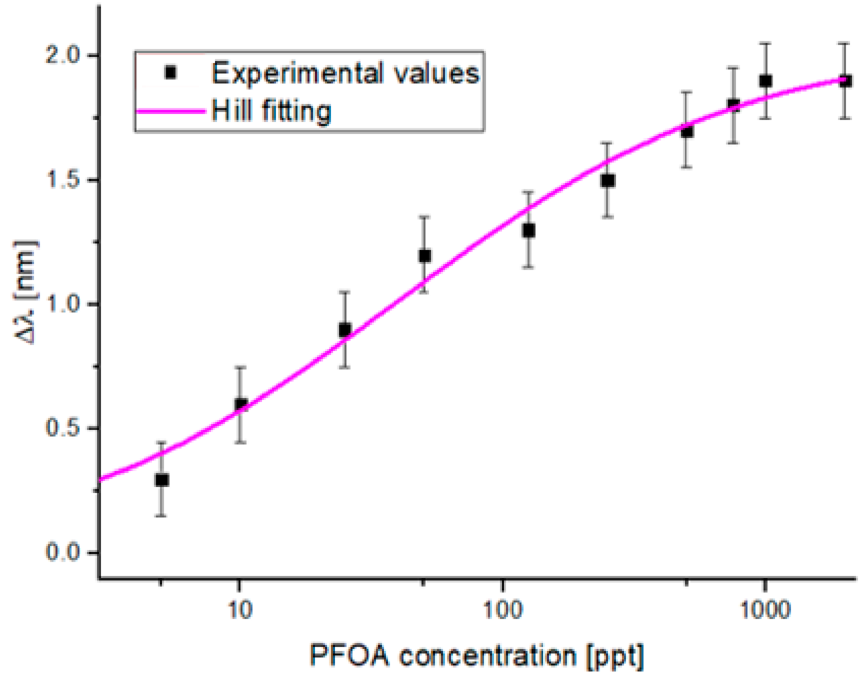

Figure S1. Resonance wavelength variations (calculated with respect to the blank) versus PFOA concentration in Milli-Q water with Hill fitting of the experimental values and error bars.

Table S1. Hill fitting parameters relative to PFOA detection.

$\Delta\lambda_0$ [nm].		$\Delta\lambda_{max}$ [nm]		K [ppt]		n		Statistics	
**Value**	**St. Error**	**Value**	**St. Error**	**Value**	St. Error	Value	St. Error	Value	St. Error
−0.025	0.077	2.056	0.126	40.097	10.972	0.652	0.100	0.275	0.985

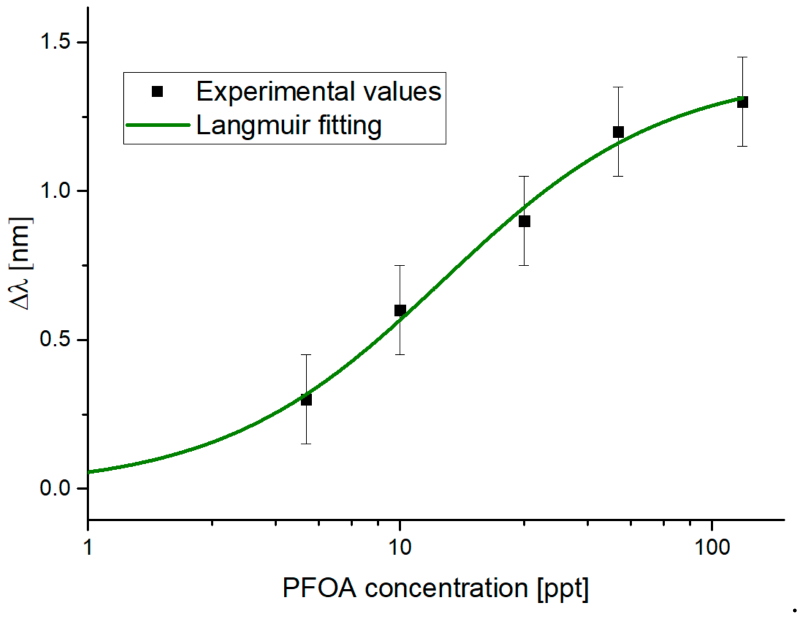

Figure S2. Resonance wavelength variations (calculated with respect to the blank) versus PFOA concentration in Milli-Q water with Langmuir fitting of the experimental values and error bars, relative to sites1 (strong).

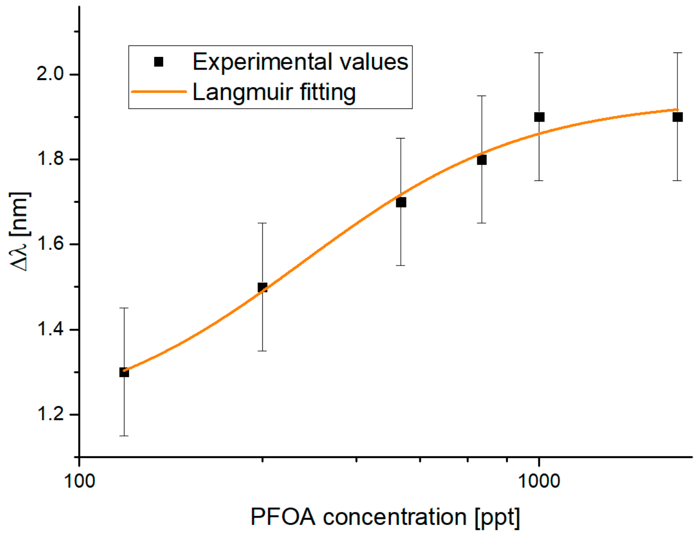

Figure S3. Resonance wavelength variations (calculated with respect to the blank) versus PFOA concentration in Milli-Q water with Langmuir fitting of the experimental values and error bars, relative to sites2 (weak).

Table S2. The Langmuir fitting parameters relative to two different sites for PFOA detection.

Type of sites	$\Delta\lambda_0$ [nm]		$\Delta\lambda_{max}$ [nm]		K [ppt]		Statistics	
	Value	St. Error	Value	St. Error	Value	St. Error	Value	St. Error
Sites1 (strong)	−0.002	0.050	1.401	0.093	13.70	2.330	0.114	0.990
Sites2 (weak)	1.174	0.155	1.942	0.058	306.512	78.095	0.054	0.978
